# Calmodulin Affects Sensitization of *Drosophila melanogaster* Odorant Receptors

**DOI:** 10.3389/fncel.2016.00028

**Published:** 2016-02-12

**Authors:** Latha Mukunda, Fabio Miazzi, Vardanush Sargsyan, Bill S. Hansson, Dieter Wicher

**Affiliations:** Department of Evolutionary Neuroethology, Max Planck Institute for Chemical EcologyJena, Germany

**Keywords:** insect olfaction, odorant receptor, *Drosophila melanogaster*, Orco, calmodulin, sensitization

## Abstract

Flying insects have developed a remarkably sensitive olfactory system to detect faint and turbulent odor traces. This ability is linked to the olfactory receptors class of odorant receptors (ORs), occurring exclusively in winged insects. ORs form heteromeric complexes of an odorant specific receptor protein (OrX) and a highly conserved co-receptor protein (Orco). The ORs form ligand gated ion channels that are tuned by intracellular signaling systems. Repetitive subthreshold odor stimulation of olfactory sensory neurons sensitizes insect ORs. This OR sensitization process requires Orco activity. In the present study we first asked whether OR sensitization can be monitored with heterologously expressed OR proteins. Using electrophysiological and calcium imaging methods we demonstrate that *D. melanogaster* OR proteins expressed in CHO cells show sensitization upon repeated weak stimulation. This was found for OR channels formed by Orco as well as by Or22a or Or56a and Orco. Moreover, we show that inhibition of calmodulin (CaM) action on OR proteins, expressed in CHO cells, abolishes any sensitization. Finally, we investigated the sensitization phenomenon using an *ex vivo* preparation of olfactory sensory neurons (OSNs) expressing Or22a inside the fly's antenna. Using calcium imaging, we observed sensitization in the dendrites as well as in the soma. Inhibition of calmodulin with W7 disrupted the sensitization within the outer dendritic shaft, whereas the sensitization remained in the other OSN compartments. Taken together, our results suggest that CaM action is involved in sensitizing the OR complex and that this mechanisms accounts for the sensitization in the outer dendrites, whereas further mechanisms contribute to the sensitization observed in the other OSN compartments. The use of heterologously expressed OR proteins appears to be suitable for further investigations on the mechanistic basis of OR sensitization, while investigations on native neurons are required to study the presently unknown additional mechanisms involved in OSN sensitization.

## Introduction

For insects the sense of smell plays a major role for orchestrating behavioral tasks such as finding food or mating partners or to avoid predators. Flying insects have to navigate in highly diluted and dispersed odor plumes as well as in regions with high odor concentrations when approaching the odor source. Such variable sensitivity of the olfactory system can be achieved by coexpression of olfactory receptor sets with different detection threshold or by regulating the receptor sensitivity according to requirements. Insect olfactory sensory neurons (OSNs) express two main families of receptor proteins, the odorant receptor (OR) and gustatory receptor (GR) proteins showing a 7-transmembrane topology like metabotropic receptors and the ionotropic receptors (IRs, Benton et al., 2009) which are related to ionotropic glutamate receptors (see reviews by Touhara and Vosshall, 2009; Kaupp, 2010; Joseph and Carlson, 2015). Interestingly, ORs evolved in parallel with the onset of insect flight and they only occur in flying insects (Missbach et al., 2014). ORs form ligand-gated ion channels immediately activated by odorant binding to the receptor complex (Sato et al., 2008; Wicher et al., 2008). They are heterodimers composed of an odorant-specific OrX protein and an ubiquitous coreceptor (Orco), both of which contribute to the ion channel pore and determine characteristics such as ion permeability (Nichols et al., 2011; Pask et al., 2011; Nakagawa et al., 2012). In addition, the ORs are controlled by intracellular signaling (Kain et al., 2008; Wicher et al., 2008; Deng et al., 2011; Sargsyan et al., 2011; Getahun et al., 2013) and the sensitivity of ORs—but not of IRs—can be adjusted by metabotropic autoregulation (Getahun et al., 2013). Stimulation of OSNs with subthreshold odor concentrations can elicit a superthreshold response when the stimulus is repeated in a suitable time window; this phenomenon is called sensitization.

Sensitization of ORs requires Orco activation (Getahun et al., 2013). For a recent review on the role of Orco in OR function see (Stengl and Funk, 2013). Heterologously expressed Orco proteins form ion channels (Wicher et al., 2008) which can be activated by synthetic agonists (Jones et al., 2011; Chen and Luetje, 2012). In previous experiments we have seen that the Orco response to synthetic agonist stimulation was modified by calmodulin (CaM) function (Mukunda et al., 2014). We observed that CaM regulation on Orco was an inherent property of the channel, due to a conserved putative CaM binding site laying on the second intracellular loop of the protein (Mukunda et al., 2014); the function of this regulatory system is still unknown. Since Orco plays a central role in ORs sensitization, modulators of Orco activity may also affect this process.

The present study is aimed to find correlates to sensitization in heterologously expressed OR proteins and in native *D. melanogaster* OSNs to investigate the molecular basis of this phenomenon. To do so, we first asked whether the Orco function was modified by repeated weak stimulation using the patch clamp technique to register the currents passing through Orco channels. As these channels permeate cations including Ca^2+^, we also performed Ca^2+^ imaging experiments without affecting the cell integrity. We thus asked whether CaM might play a role in the process of OR sensitization. We blocked CaM using both a pharmacological and a genetic approach, expressing an Orco construct bearing a point mutation (K339N) in its putative CaM binding site. Then, we investigated two heteromeric constructs, Or22a/Orco and Or56a/Orco, to test whether they could be sensitized and whether CaM inhibition could affect the sensitization of these constructs. Finally, we developed a protocol to sensitize *D. melanogaster* OSNs in *ex vivo* conditions and we monitored how sensitization in different regions of these neurons was affected by a pharmacological inhibition of CaM. In this way investigating the role of CaM on OR sensitization as a study case, we could test to which extent heterologous OR expression can represent the function of native *Drosophila* OSNs for this specific purpose.

## Materials and methods

### Cell culture and calcium imaging

CHO cells stably expressing Orco and FACS (Fluorescent activated cell sorting) CHO cells were purchased from cytobox UG (Konstanz, Germany) and grown in cytobox™ CHO select medium containing puromycin. The cells were grown on poly-L-lysine (0.01%, Sigma-Aldrich, Steinheim, Germany) coated coverslips and cultured at a density of ~2–5 × 10^5^ per four 35 mm dish. The Or22a, Or56a receptors and the OrcoCaM construct (OrcoK339N mutant, Mukunda et al., 2014) were transfected at a 0.3–0.5 μg/well-concentration using X-treme GENE HP (Roche diagnostics, Mannheim, Germany) and Rotifect transfection kit (Roth, Karlsruhe, Germany).

For calcium imaging, CHO cells were incubated in bath solution containing 5 μM fura-2/acetomethylester (Molecular Probes, Invitrogen) for 30 min. Excitation of fura-2 at 340 and 380 nm was performed with a monochromator (Polychrome V, T.I.L.L. Photonics, Gräfelfing, Germany) coupled via an epifluorescence condenser into an Axioskop FS microscope (Carl Zeiss, Jena, Germany) with a water immersion objective (LUMPFL 40xW/IR/0.8; Olympus, Hamburg, Germany). Emitted light was separated by a 400 nm dichroic mirror and filtered with a 420 nm long-pass filter. Free intracellular Ca^2+^ concentration ([Ca^2+^]_i_) was calculated according to the equation ${\left[Ca^{2+}\right]}_{i}=K_{eff}\frac{R-R_{min}}{R_{max}-R}$. *K*_*eff*_, *R*_min_, and *R*_max_ were determined as mentioned in Mukunda et al. (2014). Fluorescence images were acquired using a cooled CCD camera controlled by TILLVision 4.0 software (T.I.L.L. Photonics). The resolution was 640 × 480 pixels in a frame of 175 × 130 μm (40x/IR/0.8 objective). Image pairs were obtained by excitation for 150 ms at 340 and 380 nm; background fluorescence was subtracted.

VUAA1 (50–100 μM) and the CaM inhibitors W7 (10 μM) and CPZ (20 μM) were manually added via pipette (100 μl) with an interval of 75 s. Cells were continuously perfused with bath solution in the recording/perfusion chamber (RC-27, Warner Instruments Inc., and Hamden, CT, USA). For experimental cells the standard extracellular solution (SES) contained: 135 mM NaCl, 5 mM KCl, 1 mM CaCl_2_, 1 mM MgCl_2_, 10 mM HEPES, 10 mM glucose; pH = 7.4; osmolarity = 295 mOsmol/l.

### Electrophysiology

Ion currents in Orco expressing FACS-CHO cells were measured with the patch clamp technique in the whole-cell configuration using an EPC10 patch-clamp amplifier controlled by the Patchmaster software (HEKA, Elektronik, Lambrecht, Germany). Experiments were performed at room temperature, series resistance, and capacitive currents were compensated. Pipettes were made from borosilicate glass and had resistances of 2–4 MΩ. The pipette solution contained 140 mM KCl, 4 mM NaCl, 2.2 mM CaCl_2_, 2 mM Mg-ATP, 0.05 mM Na-GTP, 5 mM EGTA, 10 mM HEPES (pH 7.3); the bath solution was composed as described above. The pipette solution for comparing the buffering effect of EGTA vs. BAPTA contained 50 nM free Ca^2+^ adjusted with the metal/chelator ratio 1 mM CaCl_2_, 5 mM BAPTA, and 1.6 mM CaCl_2_, 5 mM EGTA (http://web.stanford.edu/~cpatton/webmaxc/webmaxcE.htm). The agonist VUAA1 (100 μM) was applied for 1 s with interval of 60 s under continuous perfusion. For agonist application the pneumatic picopump PV830 (World precision Instruments, USA) was used; cells were continuously perfused with bath solution in the recording/perfusion chamber (RC-27, Warner Instruments Inc., Hamden, CT, USA).

### Fly preparation and calcium imaging in olfactory neurons

*Drosophila melanogaster* with genotype *w, UAS-GCamP3.0;* +; *Or22a-Gal4* were maintained on conventional cornmeal agar medium under a 12 h light: 12 h dark cycle at 25°C. UAS-GCamP3.0 parental line was obtained from Bloomington Stock Center (#32234), Or22a-Gal4 line was kindly provided by Dr. Leslie Vosshall, Rockefeller University. Flies were prepared as described in Mukunda et al. (2014). Briefly, flies were anesthetized in ice and decapitated. Antennae were excised, fixed in vertical position on a glass coverslip using a two component silicon and immersed in *Drosophila* Ringer solution (5 mM HEPES, 150 mM NaCl, 5 mM KCl, 2 mM MgCl_2_, 2 mM CaCl_2_, 36 mM sucrose, pH = 7.3; osmolarity = 323 mOsmol/l). Funiculi were cut below half of their length and incubated for 5–10 min to remove possible air bubbles. Antennae were continuously perfused with Ringer solution (control) or with Ringer solution containing 10 μM W7 in the recording/perfusion chamber (RC-27, Warner Instruments Inc., Hamden, CT, USA). Stimuli, consisting each of 10 μl of 50 μM VUAA1 or ethyl hexanoate in Ringer solution, were applied manually using a Multipette Xstream (Eppendorf, Hamburg, Germany) directly into the main inflow by means of a Y connector equipped with a flow valve. The dilution factor of the stimulus inside the chamber was equivalent to a 10^−2^ factor. The time span between two subsequent stimulations was 100 s. Calcium imaging was performed as described above using a LUMPFL 60x/0.90 water immersion objective (Olympus, Hamburg, Germany). Emitted light was separated by a 490-nm dichroic mirror and filtered with a 515-nm long-pass filter. GCamP3.0 was excited with a 475 nm light at a 0.2 Hz frequency with an exposition time of 50 ms. The response magnitude was calculated for each frame as the average ΔF/F_0_ and expressed in percentage. F_0_ was estimated as the mean fluorescence level calculated for each selected region of interest as the average intensity between the 10th and the 19th frame of the recording.

### Chemicals

VUAA1 (N-(4-ethylphenyl)-2-((4-ethyl-5-(3-pyridinyl)-4H-1,2,4-triazol-3-yl)thio)acetamide) was synthesized by the working group “Mass Spectrometry/Proteomics” of the Max Planck Institute for Chemical Ecology (Jena, Germany). W7 hydrochloride was purchased from Tocris bioscience (Wiesbaden-Nordenstadt, Germany), Chlorpromazine hydrochloride (CPZ) and ethyl hexanoate (99% purity) from Sigma Aldrich (Steinheim, Germany).

### Data analysis

For electrophysiology, IgorPro (WaveMetrics, Lake Oswego, OR, USA) was used. Statistical analysis was performed with Prism 4 (GraphPad Software, Inc., La Jolla, CA, USA). Visual representation of Ca^2+^ responses in Figure 5A was obtained using Fiji (Schindelin et al., 2012) ImageJ version 2.0.0-rc-34/1.50a software, calculating ΔF/F_0_ values on a pixel basis instead than on selected regions of interest.

## Results

**Figure 1:**
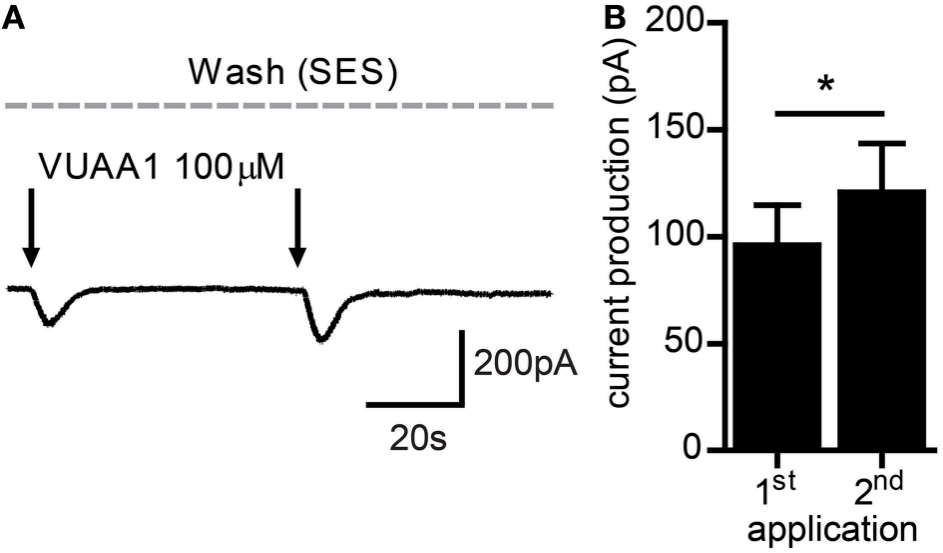
**Current flow through Orco channels increases after repeated stimulation. (A)** Representative trace of whole cell current recorded from a CHO cell expressing Orco. Currents were activated by application of the agonist VUAA1 (100 μM) (arrows). **(B)** Current amplitudes obtained as described in A (*n* = 23); mean ± SEM; paired *t*-test, ^*^p < 0.05.

As OR sensitization requires Orco activation, we first asked whether repeated weak stimulation of Orco proteins expressed in CHO cells would lead to enhanced current responses. For this sake we performed patch clamp recordings in the whole cell configuration with near-threshold stimulation of Orco using the OR agonist VUAA1. Generally, two critical parameters affect sensitization in OSNs. The first one is the time span between two stimuli. Using subthreshold odor stimulation, OR sensitization was observed for time spans between 10 s and 3 min (Getahun et al., 2013). Using a medium interval of 60 s, repeated near-threshold stimulation elicited an increased current production (Figure 1). The second one is the intensity of the first response. For stimuli eliciting a robust first response (> 300 pA) we never observed an enhanced second response. In these cases the second response was attenuated representing an adaptation (not shown).

The intracellular calcium concentration ([Ca^2+^]_i_) buffering plays an important role in regulating the Orco function (Sargsyan et al., 2011). A high degree of intracellular calcium buffering was seen to prevent Orco activation (Sargsyan et al., 2011). As the first Orco activation produces a Ca^2+^ influx and a too strong first stimulus could prevent a sensitization, we asked whether the speed of calcium buffering may affect sensitization. For a given [Ca^2+^]_i_ level of 50 nM in the pipette solution, adjusted with the slow calcium buffer EGTA (Ca^2+^ exchange rate constant = 0.4 s^−1^ at pH = 7; Hellam and Podolsky, 1969) or with fast buffer BAPTA (Ca^2+^ exchange rate constant ≥ 60 s^−1^; Tsien, 1980), we compared the amount of current amplification between these two conditions. Using BAPTA the normalized second current response was 1.82 ± 0.48 (*n* = 9), for EGTA it was 1.44 ± 0.24 (*n* = 7) which was not significantly different (unpaired *t*-test).

**Figure 2:**
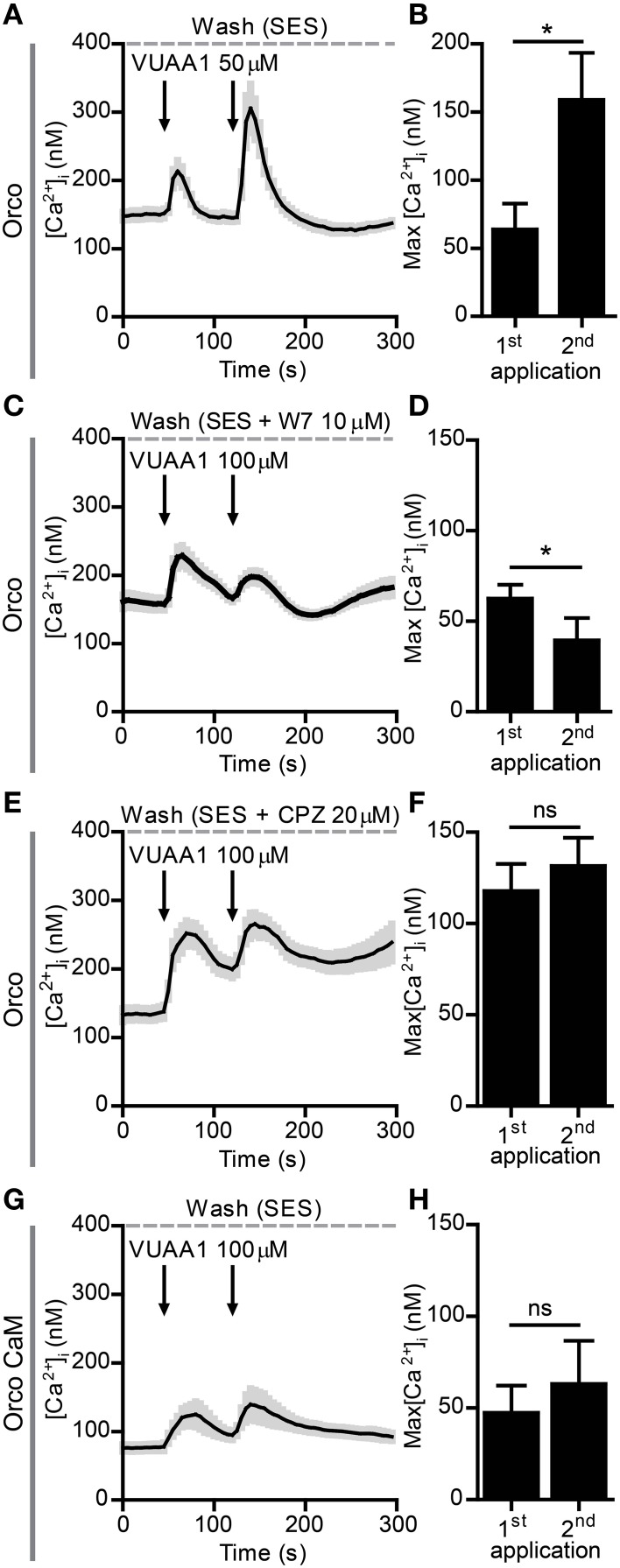
**Sensitization of Orco channels is abolished by Calmodulin (CaM) inhibition. (A,B)** Averaged recordings of [Ca^2+^]_i_ in cells expressing Orco (**A**, *n* = 14) stimulated with 50 μM VUAA1 (arrows); **(B)** Maximum increase in [Ca^2+^]_i_ after 1^st^ and 2^nd^ application of VUAA1 as in **(A)**. **(C–F)** Averaged recordings of [Ca^2+^]_i_ in cells expressing Orco in presence of the CaM inhibitors W7 (**C**, *n* = 29) and chlorpromazine (CPZ, **E**, *n* = 9) and maxima of [Ca^2+^]_i_ rise with W7 **(D)** and CPZ **(F)**. **(G,H)** Averaged recordings of [Ca^2+^]_i_ **(G)** and maxima of [Ca^2+^]_i_ rise **(H)** in cells expressing the Orco CaM mutant (*n* = 14). Data represent mean ± SEM; paired *t*-tests, ^*^p < 0.05, ns, not significant.

We next tried to observe sensitization with excised patches to determine how this phenomenon is manifested at the level of single channels. Unfortunately we got no increases in the responses, both for inside out as well as outside out configuration. The possible reason for this might be a change in the regulatory environment due to patch excision, e.g., by a dephosphorylation or a loss of parts of a signaling cascade. We thus decided to perform further investigations with the non-invasive Ca^2+^ imaging technique. In a previous study we have characterized the Ca^2+^ responses of Orco channels expressed in CHO cells when stimulated with VUAA1 (Mukunda et al., 2014). Similar to the findings from patch-clamp experiments, a repeated stimulation after a first robust response ([Ca^2+^]_i_ > 200 nM) did not induce sensitization (not shown), whereas stimulations eliciting moderate responses resulted in an amplified Ca^2+^ signal in cells expressing Orco (Figures 2A,B).

**Figure 3:**
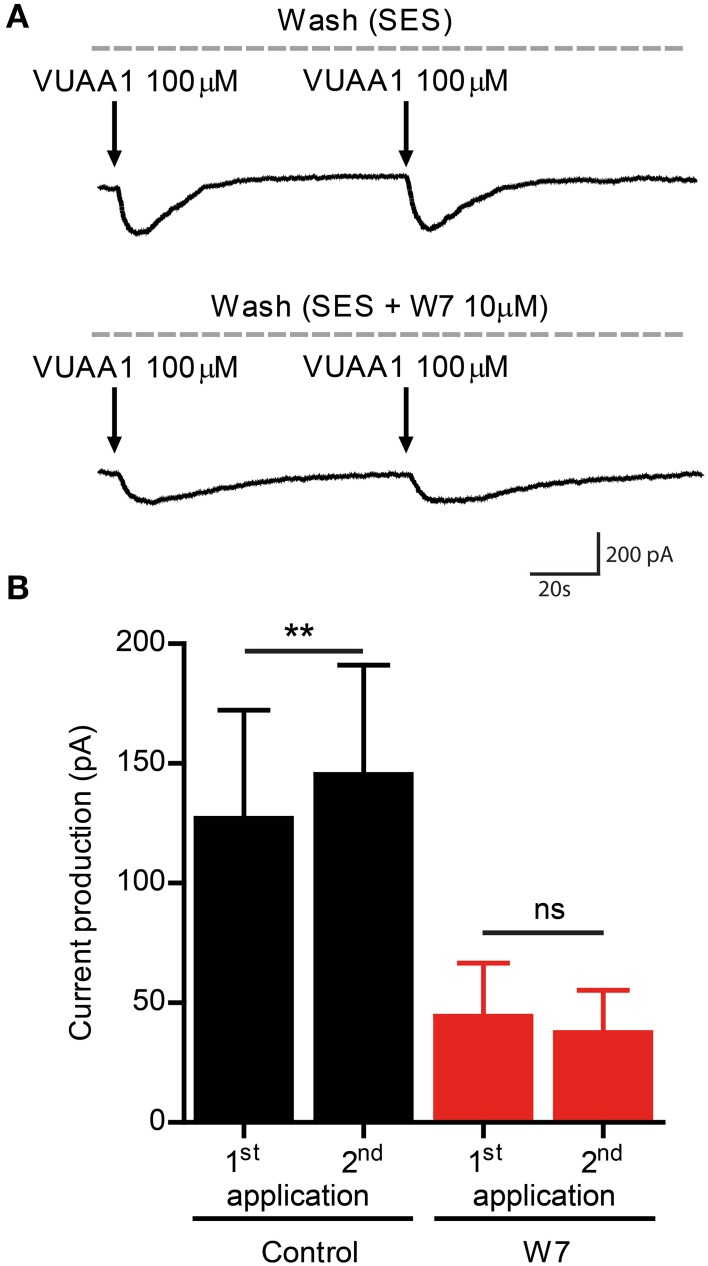
**The CaM inhibitor W7 abolishes enhanced current flow through Orco channels after repeated stimulation. (A)** Representative traces of whole cell currents recorded from a cell expressing Orco. Currents were obtained by VUAA1 application in the absence (top) and in presence of W7 (bottom). **(B)** Current amplitudes obtained as described in **(A)** (Control, *n* = 8; W7, *n* = 8). Data represent mean ± SEM; paired *t*-tests, ns, not significant, ^**^p < 0.01.

A previous study revealed a control of Orco function by CaM (Mukunda et al., 2014). Inhibition of CaM function reduced the Orco response upon VUAA1 stimulation and prolonged the response. Here we tested whether CaM might be involved in Orco sensitization. The double stimulation protocols were used in the presence of the CaM inhibitors W7 (Figures 2C,D) and chlorpromazine (CPZ) (Figures 2E,F) on Orco. The quantitative difference in the effect of W7 and CPZ may reflect the fact that these compounds have additional effects, i.e., W7 can affect the electrostatic surface potential of cells (Sengupta et al., 2007) whereas the cationic amphipath CPZ can affect the membrane tension (Lundbaek, 2008). We also tested the Orco CaM mutant bearing a point mutation in the putative CaM binding site (Figures 2G,H). Stimulation of Orco expressing cells in these conditions did not induce a response using a moderate stimulation (50 μM VUAA1, not shown). For this reason we used stimuli of 100 μM VUAA1, which ensured a consistent first response not significantly higher than the control (Supplementary Figure 1). In all cases tested there was no significant potentiation of the response at the second stimulus (Figures 2C–H). To exclude the possibility that delayed basal [Ca^2+^]_i_ recovery affects OR sensitization, we repeated the experiments with pharmacological inhibition of CaM, increasing the time span between the two stimulations from 75 to 100 s; even in these conditions no sensitization was detected (Supplementary Figure 2). To support the findings obtained on the role of CaM with Ca^2+^ imaging we performed whole-cell patch-clamp experiments. When cells having shown sensitization by repeated VUAA1 stimulation were treated with W7, a double stimulation with the same protocol did not enhance the second current response, and both stimuli elicited a general weaker response (Figures 3A,B). Thus, both Ca^2+^ imaging and electrophysiological experiments support the view that CaM activity is required to establish sensitization in heterologously expressed Orco proteins.

**Figure 4:**
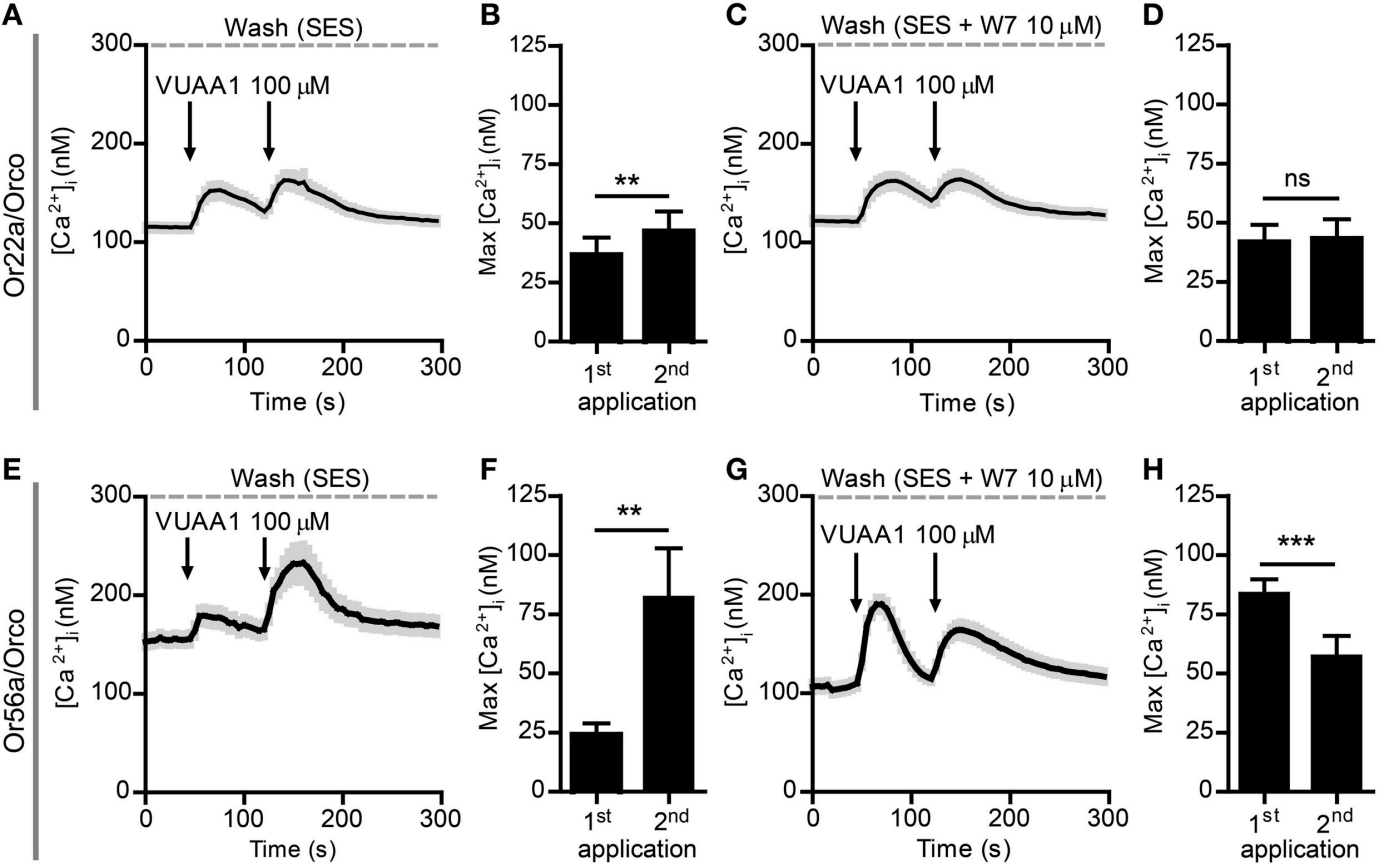
**Sensitization of Or22a/Orco and Or56a/Orco constructs is abolished by the CaM inhibitor W7. (A–D)** Averaged recordings of [Ca^2+^]_i_ in cells expressing Or22a and Orco stimulated with 100 μM VUAA1 (arrows) in absence (**A**, *n* = 29) and in presence of W7 (**C**, *n* = 21) and maxima of [Ca^2+^]_i_ rise without **(B)** and with W7 **(D)**. **(E–H)** Averaged recordings of [Ca^2+^]_i_ in cells expressing Or56a and Orco stimulated with 100 μM VUAA1 (arrows) in absence (**E**, *n* = 19) and in presence of W7 (**G**, *n* = 52) and maxima of [Ca^2+^]_i_ rise without **(F)** and with W7 **(H)**. Data represent mean ± SEM, paired *t*-tests, ns, not significant, ^**^p < 0.01, ^***^p < 0.001.

Next, we tested two complete OR constructs which were previously seen to differ in their responses to CaM inhibition, namely Or22a/Orco and Or56a/Orco (Mukunda et al., 2014). In order to discriminate the cell populations expressing Or22a/Orco and Or56a/Orco from those expressing Orco alone we determined the distribution of the [Ca^2+^]_i_ signal decay time constants τ that we have previously seen to vary between cells transfected with Orco alone or with an OrX/Orco complex (Mukunda et al., 2014). We set threshold values to separate them as shown in Supplementary Figure 3. Under control conditions, the τ distributions were composed of two clusters, one of which representing cells expressing Orco alone and the other one representing cells expressing both OR proteins (Supplementary Figures 3A–C). In the presence of W7 the τ distribution of cells expressing Orco became broader and shifted toward higher τ-values, the threshold was thus shifted accordingly (Supplementary Figures 3D–F). In cells expressing complete ORs the second VUAA1 stimulation amplified the first Ca^2+^ response elicited under control conditions (Figures 4A,B,E,F) but not in presence of 10 μM W7 (Figures 4C,D,G,H). We also tried to sensitize the Or22a/Orco construct by stimulation with the Or22a ligand ethyl hexanoate (100 μM) and observed two different patterns of responses. In fact, when the first stimulus gave rise to a Ca^2+^ response comparable to those elicited by VUAA1 to get an enhanced second response, the second pulse always failed to enhance the Ca^2+^ signal (Supplementary Figure 4A) but when the first stimulus failed to produce a Ca^2+^ signal, there was a response upon the second stimulus (Supplementary Figure 4B) which could be interpreted as result of OR sensitization according to Getahun et al. (2013). In both cases the preparation does not provide a sufficiently controlled system to test the role of CaM on OR sensitization.

**Figure 5:**
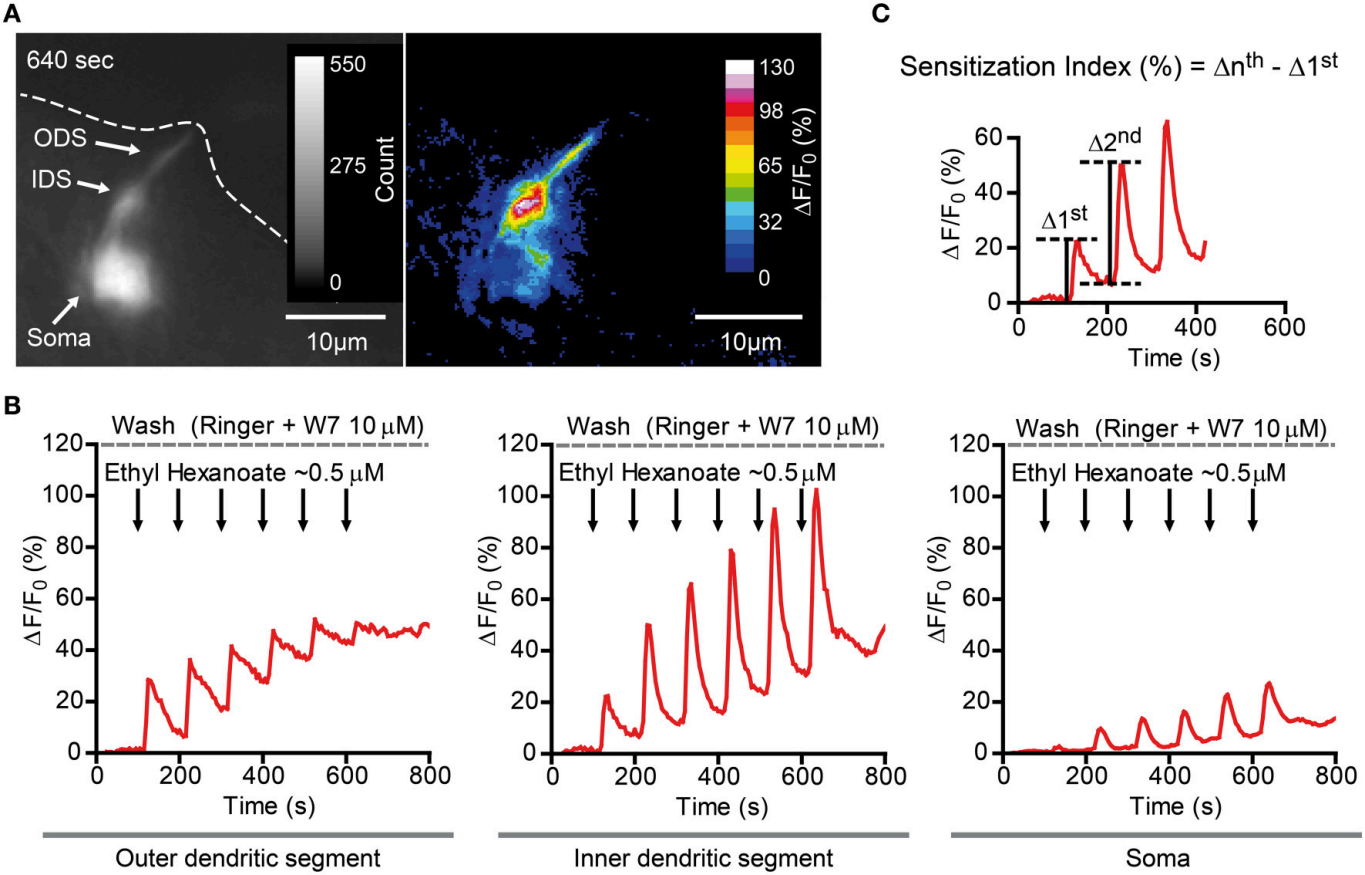
**Ca^2+^ imaging example of a *D. melanogaster* Or22a (and GCaMP3.0) expressing OSN in presence of 10 μM W7. (A)** Left: GCaMP fluorescence intensity of an OSN inside the fly antenna. Note the soma (Soma), the inner dendritic segment (IDS) and the outer dendritic segment (ODS), which reaches the rim on the antennal cut (dashed line). The figure represents a frame captured at 640 s (peak of the last stimulation). Right: ΔF/F_0_ (percent) of the frame in the left panel. **(B)** Plot of fluorescence intensity over time of different compartments of the same neuron as in **(A)**, namely the ODS (left), the IDS (middle), and the soma (right). **(C)** Sensitization was quantitatively assessed measuring the here called “Sensitization Index” (percent). The intensity of the *n*^th^ stimulation (Δ*n*^th^) was evaluated after subtraction of the baseline fluorescence intensity before the delivery of the *n*^th^ stimulation. The Sensitization Index was therefore calculated as the difference between the intensity of the *n*^th^ stimulation and the first stimulation (Δ*n*^th^ -Δ 1^st^) and is therefore independent of the different baseline levels between these two stimulations.

**Figure 6:**
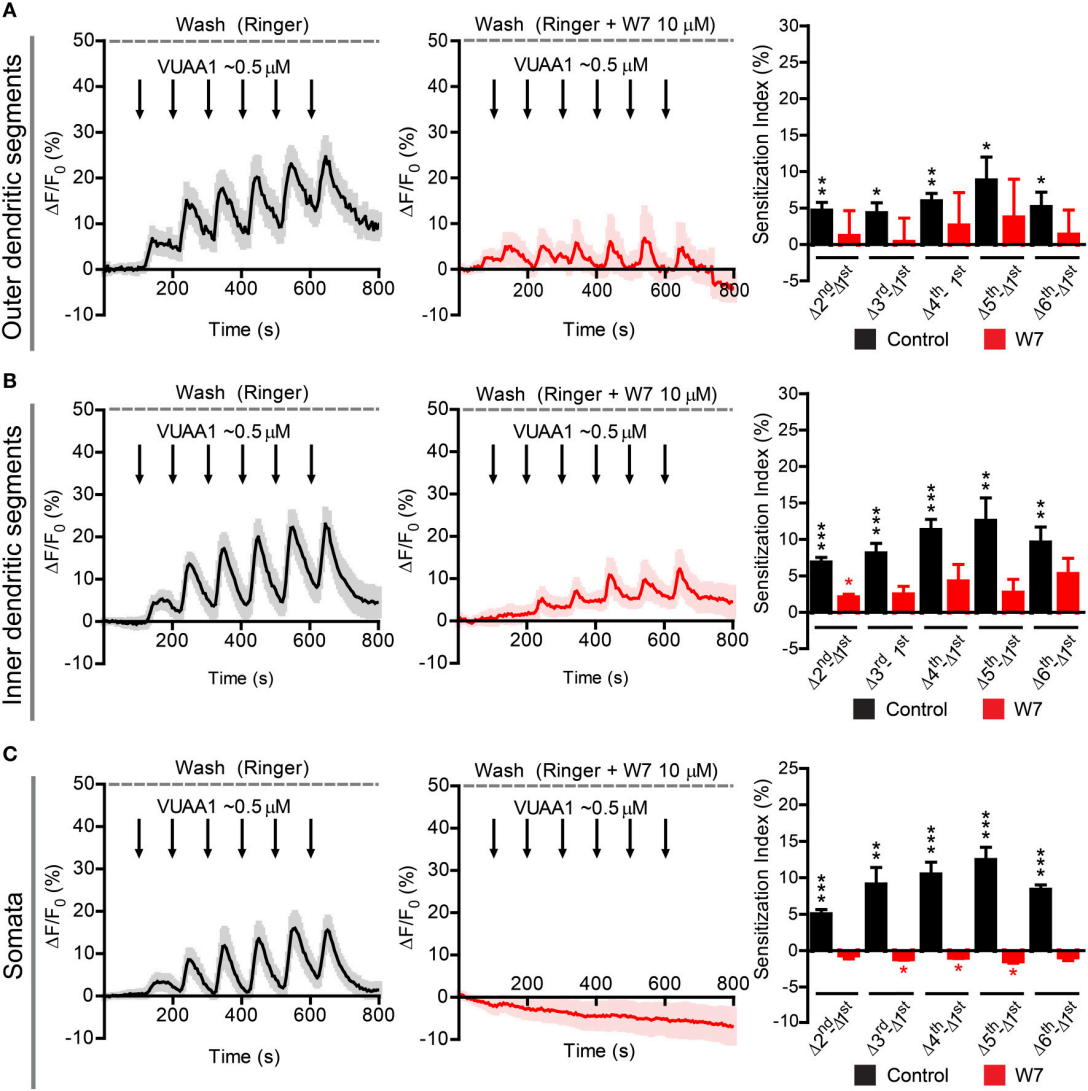
**Sensitization in different compartments of Or22a OSNs stimulated with VUAA1 in absence or presence of the strong calmodulin inhibitor W7**. ΔF/F_0_ (%) over time recorded in control conditions (left panels, black) and in presence of 10 μM W7 (middle panels, red) in the: **(A)** ODSs of the neurons (left, *n* = 8 from 5 antennae; middle *n* = 3 from 3 antennae), **(B)** IDSs of the neurons (left, *n* = 12 from 7 antennae; middle, *n* = 4 from 2 antennae), **(C)** Somata of the neurons (left, *n* = 17 from 6 antennae; middle, *n* = 4 from 4 antennae). Right panels show the Sensitization Index (%), calculated as described in Figure 5C, in control conditions (black) and in presence of W7 (red) quantified for the Outer dendritic segments **(A)**, the Inner dendritic segments **(B)**, and the Somata **(C)**; one-sample *t*-tests (μ_0_ = 0, representing no sensitization event), ^***^p < 0.001, ^**^p < 0.01, ^*^p < 0.05. All graphs represent mean ± SEM values.

**Figure 7:**
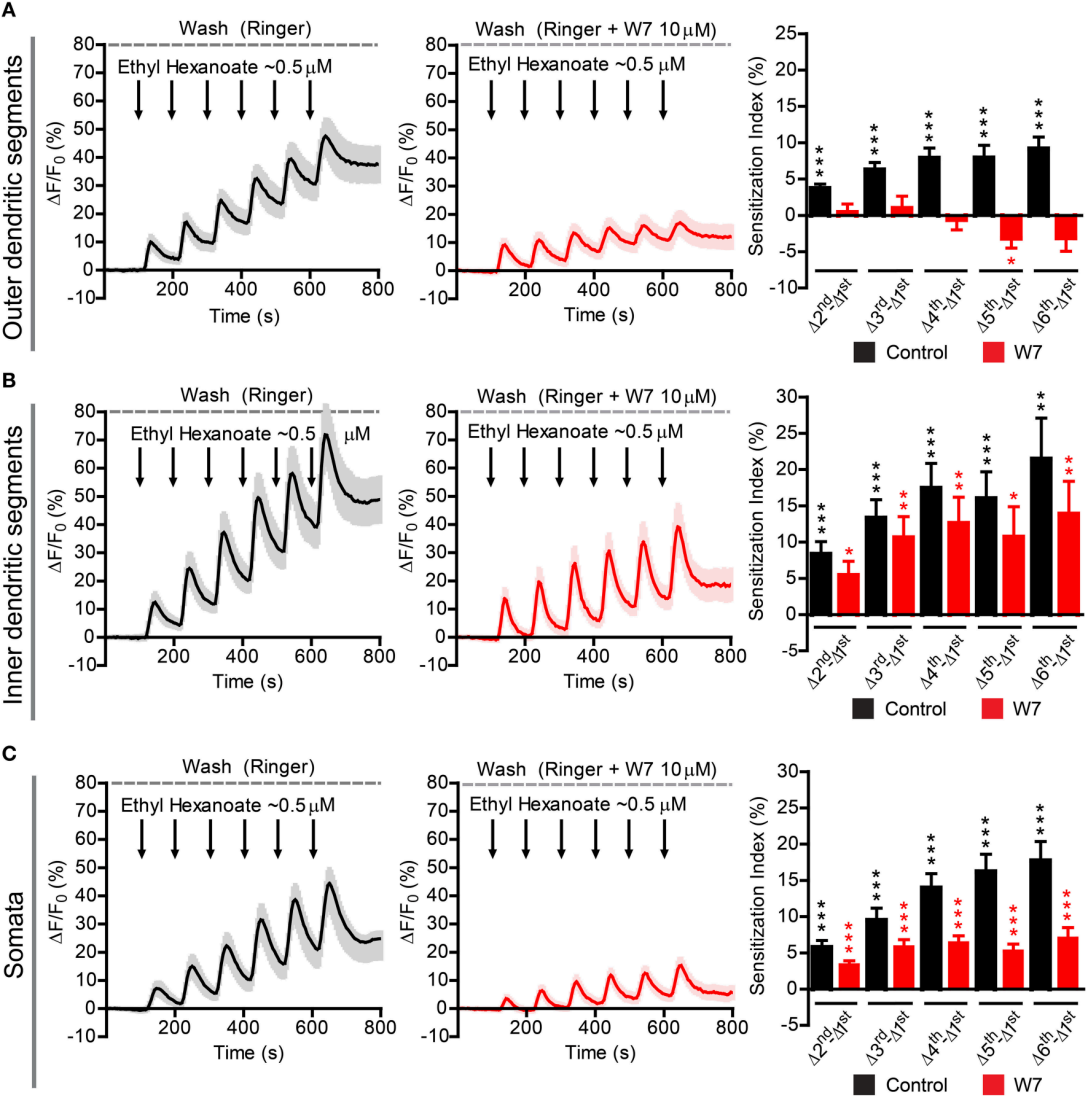
**Sensitization in different compartments of Or22a OSNs stimulated with ethyl hexanoate in absence or presence of the strong calmodulin inhibitor W7**. ΔF/F_0_ (%) over time recorded in control conditions (left panels, black) and in presence of 10 μM W7 (middle panels, red) in the: **(A)** ODSs of the neurons (left, *n* = 16 from 5 antennae; middle *n* = 15 from 5 antennae), **(B)** IDSs of the neurons (left, *n* = 21 from 7 antennae; middle, *n* = 13 from 5 antennae), **(C)** Somata of the neurons (left, *n* = 32 from 10 antennae; middle, *n* = 17 from 7 antennae). Right panels show the Sensitization Index (%), calculated as described in Figure 5C, in control conditions (black) and in presence of W7 (red) quantified for the Outer dendritic segments **(A)**, the Inner dendritic segments **(B)**, and the Somata **(C)**; one-sample *t*-tests (μ_0_ = 0, representing no sensitization event), ^***^p < 0.001, ^**^p < 0.01, ^*^p < 0.05. All graphs represent mean ± SEM values.

Finally, we asked whether the findings obtained in the heterologous expression system are representative for processes taking place in native *D. melanogaster* OSNs. For this purpose we performed Ca^2+^ imaging in *ex vivo* olfactory neurons expressing the Or22a odorant receptor and we repeatedly stimulated the preparation with submicromolar concentrations of VUAA1 (**Figure 6**) and ethyl hexanoate (**Figure 7**). A 60x objective allows to observe [Ca^2+^]_i_ dynamics within distinct cellular compartments, i.e., the soma, the outer dendritic segment (ODS) entering the sensillum and the inner dendritic segment (IDS) in between (Figures 5A,B; Supplementary Video). In order to quantify sensitization in these neurons, we subtracted the intensity of the first response from that of subsequent stimulations, after base level fluorescence intensity subtraction, and we refer to this measure as “sensitization index” (Figure 5C). We then compared the sensitization index elicited by subsequent stimulations in different areas of the neurons in presence of 10 μM W7 in the Ringer solution used for perfusion or in control conditions. Stimulation with VUAA1 in control conditions led to sensitization of all three compartments, while in presence of W7 a residual sensitization was detected in the IDSs (Figure 6B), but not in the ODSs and the somata (Figures 6A,C); on the other hand, after stimulation with ethyl hexanoate somata and IDSs showed sensitization in both experimental conditions (Figures 7B,C), while ODSs showed a net sensitization in control conditions but not in presence of W7 (Figure 7A), showing instead adaptation.

## Discussion

The regulation of the neuronal response according to the flow of sensory input forms the basis of learning and memory and is necessary to organize an appropriate behavioral response of an organism. The time domain this regulation spans may vary from milliseconds to weeks as shown in the classical example of the *Aplysia* siphon withdrawal reflex (Pinsker et al., 1973). In insects, for example, a short exposure to sex pheromones can increase the sensitivity for such pheromones in both, the short-term as well as in the long-term scale (Anderson et al., 2007). While such regulations often include changes on the level of neuronal networks, we here investigate a regulation that takes place at the level of sensory receptor proteins. Using extracellular recording of *Drosophila* OSNs we have recently shown that Orco activation is important for the regulation of insect OR sensitivity. Repetitive subthreshold odor stimulations elicited sensitized OR responses in the OSNs (Getahun et al., 2013). In the present study we asked whether the sensitization phenomenon in OSNs is based on the sensitization of ORs and could be observed in heterologously expressed OR proteins. Indeed, repeated VUAA1 stimulation of Orco expressed in CHO cells induced an increase in the response. This sensitization phenomenon was observed both in electrophysiology recordings as well as calcium imaging experiments (Figures 1, 2A,B).

CaM modulates the response of Orco channels to stimulation with VUAA1 (Mukunda et al., 2014). In the present study we could show that CaM inhibition abolished any sensitization in cells expressing Orco alone or together with the OrX proteins Or22a and Or56a (Figures 2–4). Orco contains a putative CaM binding motif which is conserved across insect species (Mukunda et al., 2014). Similar to application of CaM inhibitors, repeated stimulation of cells expressing Orco CaM mutant (K339N) failed to show a sensitization process (Figures 2G,H).

These results support the hypothesis that sensitization is an intrinsic property of *Drosophila* ORs, depending on the amino acid sequence and on interaction with downstream signaling cascades including CaM (but cf. below). On the other hand, our results from native *Drosophila* OSNs clearly show that, at least in Or22a expressing neurons, sensitization of ORs and sensitization of OSNs are not the same. Stimulation of Or22a/Orco and Orco/Orco complexes using VUAA1 induced sensitization of all three identified cellular compartments in control conditions, while we could detect a residual sensitization only in IDSs in presence of W7 (Figure 6). Exclusive stimulation of Or22a/Orco complexes with ethyl hexanoate led to the sensitization of all three compartments in control conditions, but W7 application abolished this phenomenon only in ODSs. Moreover, despite of a reduced Ca^2+^ influx in the dendritic region, we still observed increasing responses in other compartments, namely the soma and the inner dendritic segment where this phenomenon was most pronounced (Figure 7). We speculate that a possible explanation for these observations can lay in the cell's morphology and in a different design of signal transduction cascades.

**Figure 8:**
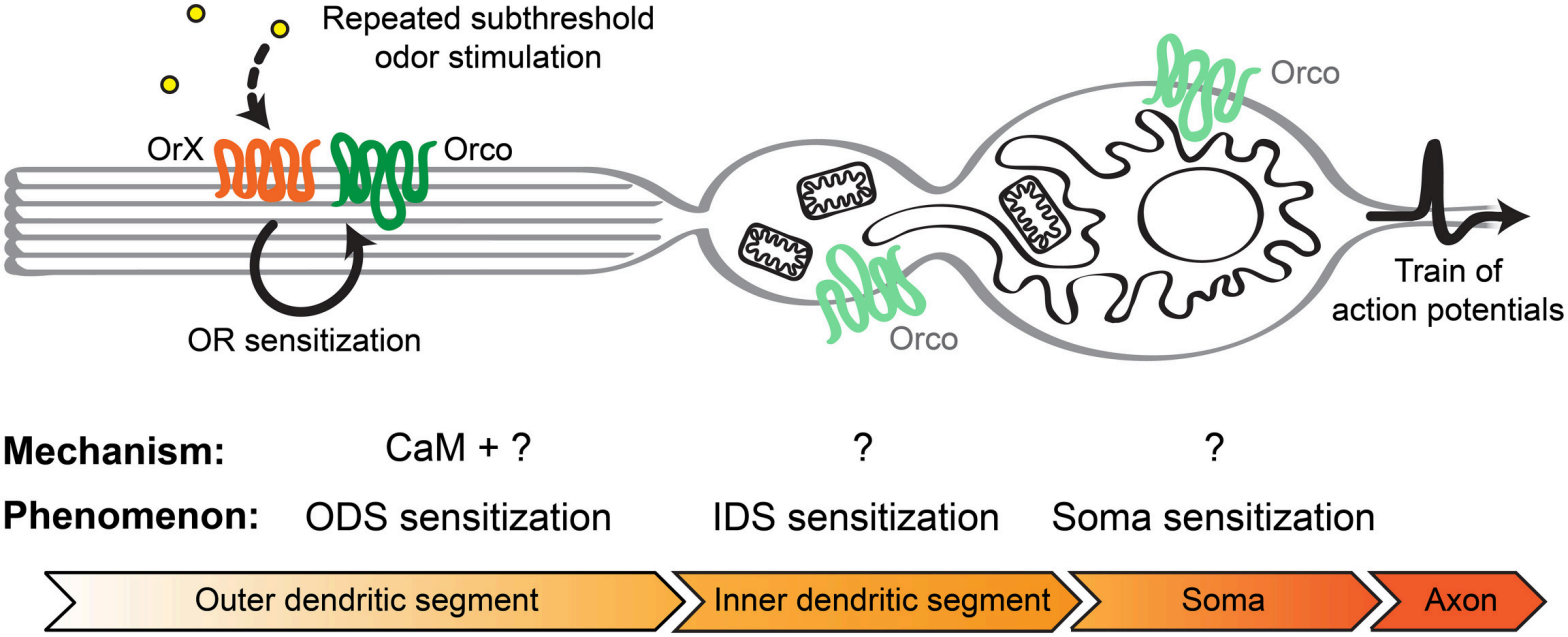
**Model to explain insect olfactory neuron sensitization in basiconic sensilla**. Following repeated stimulation with subthreshold odor concentrations of the OrX/Orco receptor complex in the olfactory cilia of the outer dendritic segment, CaM together with other unknown intracellular or membrane factors can lead to OR sensitization. This may lead to the observed ODS sensitization. Small currents originating from the ODS might trigger Ca^2+^ release from intracellular Ca^2+^ stores, including mitochondria and the endoplasmatic reticulum, first in the IDS and subsequently in the soma. In this way these cellular compartments become sensitized and the signal becomes more and more amplified. Finally, the membrane potential at the axon hillock reaches the action potentials threshold faster which enhances the firing rate.

Insect olfactory neurons are highly polarized cells. They present at the two opposite poles of the cell an axonal process and a single dendritic process terminating in multiple olfactory cilia. Orco and the neuron-specific OrX proteins only occur in the ciliar region of OSNs, while Orco proteins are broadly distributed throughout the cell (Larsson et al., 2004). Between the outer dendritic region composed of the olfactory cilia and the soma there is a specialized structure called inner dendritic segment (Shanbhag et al., 2000). This region is enriched with elongated mitochondria in trichoid and basiconic (housing e.g., Or22a expressing OSNs) sensilla, but not in coeloconic sensilla (Shanbhag et al., 2000). Moreover, *D. melanogaster* OSNs localized in basiconic and trichoid sensilla present a well-developed smooth endoplasmatic reticulum (Shanbhag et al., 2000). Therefore, the increased Ca^2+^ response in the inner dendritic segments and somata of Or22a neurons may be related to Ca^2+^ release from intracellular Ca^2+^ stores (Figure 8). A study performed on heterologously expressed insect ORs revealed an amplification of odor induced calcium responses through activation of intracellular Ca^2+^ release channels (Ignatious Raja et al., 2014), but this is to our knowledge the first report suggesting this possibility for native *Drosophila* OSNs as well.

Such a mechanism of signal amplification is different from that observed in other insect senses, e.g., vision, where the complex metabotropic signaling cascade occurs in the microvilli of rhabdomeres without involving other prominent morphological compartments of these cells (Katz and Minke, 2009). Although odorant receptor structure and olfactory signaling are different between insects and vertebrates (Kaupp, 2010), commonalities in neuronal morphology between them can hint at possible common strategies to amplify signals downhill the odorant receptors. In mammals, mitochondrial Ca^2+^ mobilization has been reported to play a key role in maintaining the broad dynamic response range and the sensitivity of olfactory neurons (Fluegge et al., 2012). In addition, mitochondria were recruited in an activity-dependent manner to the dendritic knobs between the somata and the dendritic shafts (Fluegge et al., 2012). A further analysis of intracellular calcium signaling using *in vivo* studies is required as it might play a role in olfactory signaling in insects as well (Ignatious Raja et al., 2014).

However, even in the relatively simple heterologous expression system the OR sensitization mechanism is not well understood. Although we could observe this phenomenon in such system, there were clear differences regarding the stimuli, VUAA1 vs. odor (Supplementary Figure 4). It is known that VUAA1 activates both Orco alone and OrX/Orco complexes (Jones et al., 2011), while odorant binding sites are specific to OrX proteins. Moreover, Orco proteins and OrX/Orco complexes show differences regarding their biophysical properties, including ion selectivity (Jones et al., 2011; Nakagawa et al., 2012). Differences in the ion permeability or other channel properties between Or22a/Orco complexes upon activation with ethyl hexanoate vs. VUAA1 could be responsible of the response patter in presence of the odor. This point deserves special attention in further investigations.

In heterologously expressed *Drosophila* ORs, CaM does not show a generalized effect independent of the specific OrX proteins (Mukunda et al., 2014). Moreover, the effect of CaM inhibition differed qualitatively between different OrX proteins. As a consequence, we can exclude that CaM activation due to Ca^2+^ influx via Orco activation directly leads to OR sensitization. There is thus a missing link between Orco activation and OR sensitization. Recently published studies report as yet unknown mechanisms regulating the OR function. Interestingly, the dATP8B phospholipid flippase was shown to be essential for an appropriate sensitivity of ORs, but not of IRs. It has been shown that mutation in the dATP8B gene strongly reduces the sensitivity of OR-expressing neurons (Liu et al., 2014). Understanding the basis and the molecular mechanism of insect OR sensitization relies on the knowledge of further regulators of sensitivity.

Taken together, our study shows that repeated stimulation of heterologously expressed Orco caused an enhanced response, similar as observed in *Drosophila* OSNs (Getahun et al., 2013). We also provide evidence that the sensitization process in heterologously expressed ORs and in the outer dendrites of native OSNs can be abolished by CaM inhibition. This suggests that CaM may play a central role in mediating sensitization of odorant receptors, but this contribution is not sufficient to completely explain sensitization within all OSN compartments, where Ca^2+^ release from intracellular Ca^2+^ stores may be involved. Further, studies on heterologously expressed ORs will provide insights into sensitization of odorant receptors, while studies using *in vivo* and *ex vivo* OSN preparations will deepen our knowledge about the cellular machinery components involved in the olfactory transduction in native neurons. This study illustrates the value of a heterologous expression system for the study of protein function on one hand, but it also shows the restriction of such a system when it comes to understand a native cell that expresses this protein.

## Author contributions

All authors listed, have made substantial, direct and intellectual contribution to the work, and approved it for publication.

### Conflict of interest statement

The authors declare that the research was conducted in the absence of any commercial or financial relationships that could be construed as a potential conflict of interest.
