# Supplementary material for: Calmodulin Affects Sensitization of Drosophila melanogaster Odorant Receptors
Source: Front Cell Neurosci. 2016 Feb 12;10:28. doi: 10.3389/fncel.2016.00028 (PMC4751262; doi:10.3389/fncel.2016.00028)
Supplement: Supplementary file 2 [file Presentation1.PDF]

Supplementary Material

Calmodulin affects sensitization of *Drosophila melanogaster* odorant receptors

Latha Mukunda[#], Fabio Miazzi[#], Vardanush Sargsyan, Bill S. Hansson, Dieter Wicher[*]

[#] These authors contributed equally to the work

*** Correspondence:** Dieter Wicher, Max Planck Institute for Chemical Ecology, Department of Evolutionary Neuroethology, Hans-Knöll Str. 8, Jena, D-07745, Germany.

dwicher@ice.mpg.de

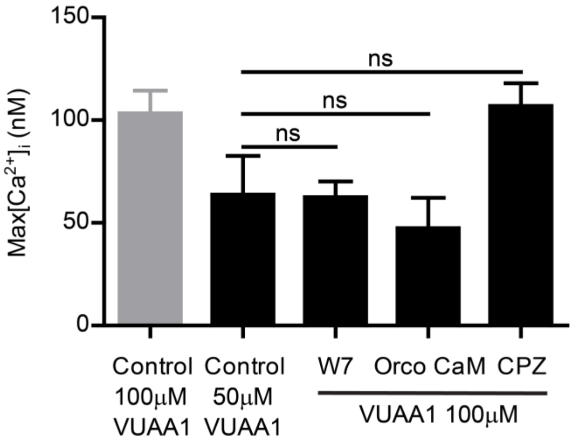

Supplementary Figure 1. Comparison of the maximum increase in $[Ca^{2+}]_i$ after the 1st stimulation in control conditions with 100 µM VUAA1 (n = 24) and 50 µM VUAA1 (n = 14) or 100 µM VUAA1 in case of pharmacological CaM inhibition with W7 (n = 29) or CPZ (n = 9) and with a point mutation on the putative CaM binding site on Orco protein (Orco CaM, n = 14). Data represent mean ± SEM, unpaired t-tests with Welch's correction in case of heteroscedasticity, ns = not significant.

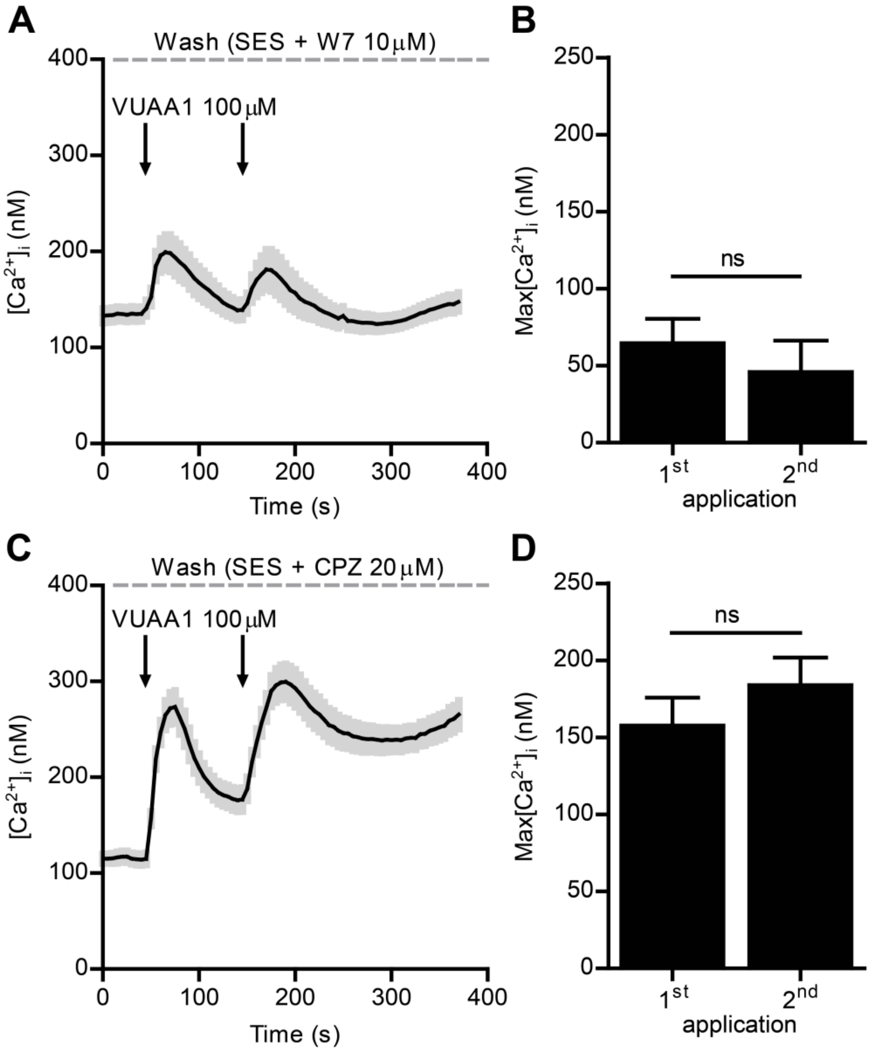

Supplementary Figure 2. Effect of CaM inhibitors on the response to VUAA1 application (100 µM) at 45 and 145 seconds in cells expressing Orco. Averaged recordings of $[Ca^{2+}]_i$ in presence of W7 (A, n = 28), chlorpromazine (CPZ, C, n = 21) and maxima of $[Ca^{2+}]_i$ rise with W7 (B) and CPZ (D). Data represent mean ± SEM, paired t-test, ns = not significant.

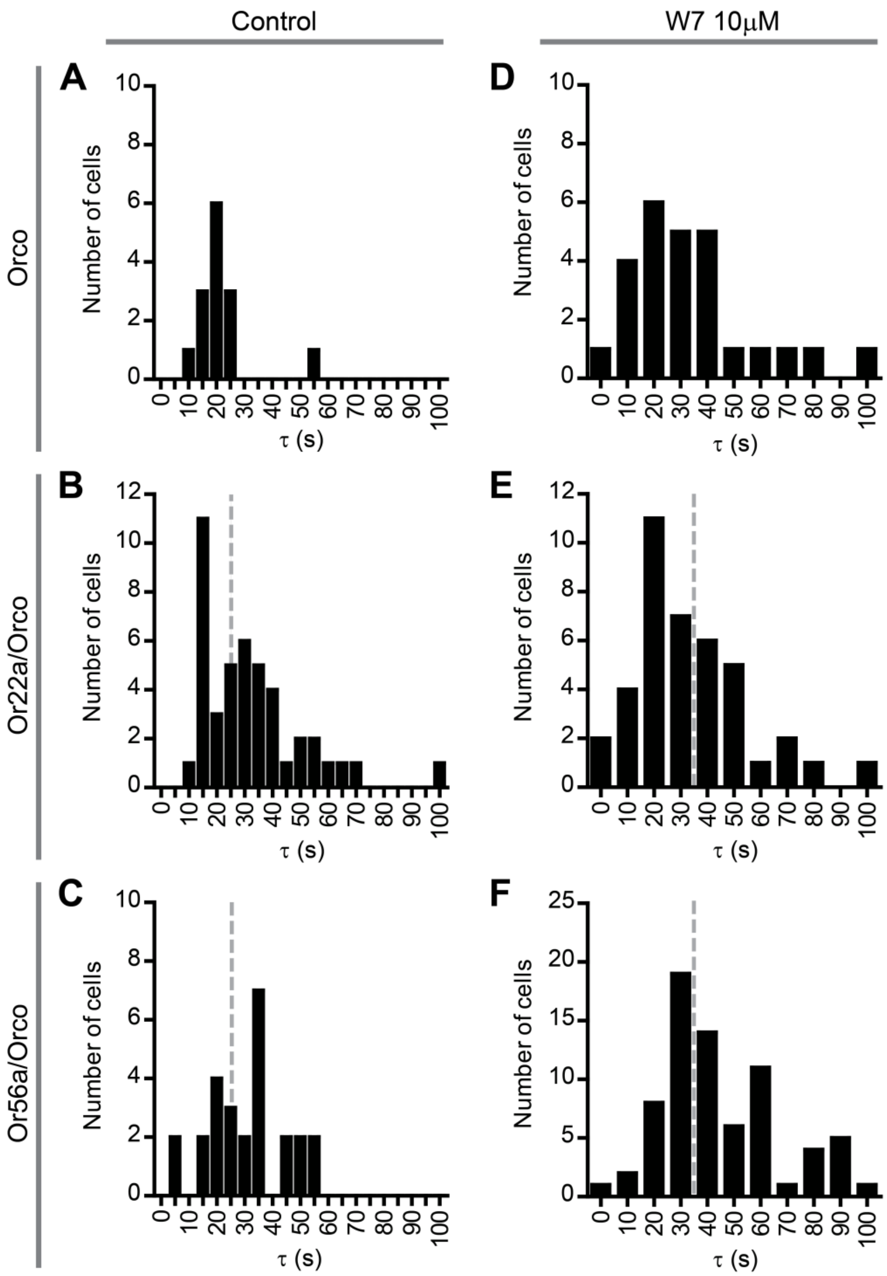

Supplementary Figure 3. Separation of cell populations putatively expressing Orco alone and Or22a/Orco or Or56a/Orco complexes by means of their distribution of the time constants of $[Ca^{2+}]_i$ decay (τ) after the second stimulation with VUAA1. (A-C) Distribution of τ values of cells in control conditions (A) expressing Orco alone – as shown in Figure 2A – calculated between 150 and 250 s, n = 14; (B) transfected with Or22a calculated between 165 and 300 s, n = 44; (C) transfected with Or56a calculated between 165 and 300 s, n = 26. Distributions in (B) and (C) show two main peaks, one between 15-20 s and the other between 30 and 35 s. In both cases the first peak is compatible with the distribution of τ values in panel A and represents cells expressing Orco alone, while the other peaks represent cells expressing an OrX/Orco complex, as shown in Mukunda et al., 2014. (D-F) Distributions of τ values of cells in presence of 10 μM W7 (D) expressing Orco alone – as shown in Figure 2C – calculated between 155 and 210 s, n = 26; (E) transfected with Or22a calculated between 155 and 300 s, n = 40; (F) transfected with Or56a calculated between 160 and 300 s, n = 72.

Distributions in (E) and (F) do not show two clear peaks due to the broader τ distribution in (D) and to the variable effect of W7 in presence of different OrX/Orco constructs (Mukunda et al, 2014). Dashed lines represent the cutoff values used to identify the cells putatively expressing Or22a/Orco and Or56a/Orco in (B) and (C) $\tau \geq 25$ s, (E) and (F) $\tau \geq 35$ s, which were used for subsequent analysis and represented in Figure 4. Cells showing a $\tau > 100$ s are not displayed here, but were used for subsequent analysis. Ticks on the x axis represent bin center values.

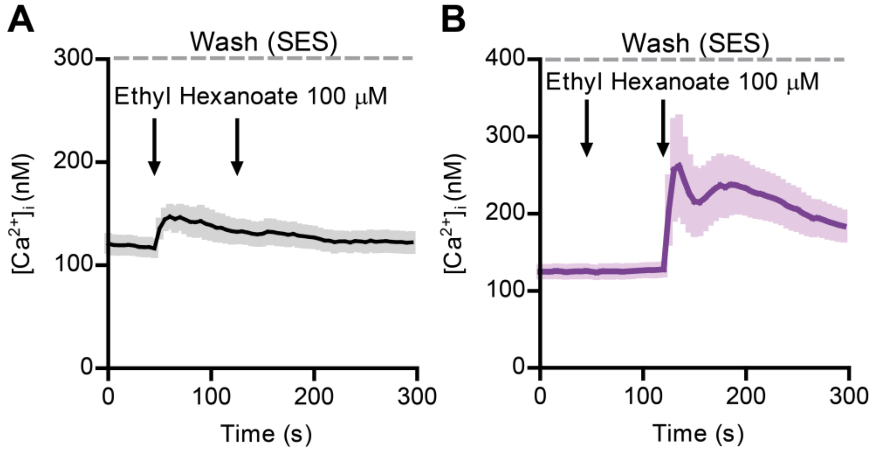

Supplementary Figure 4. Averaged recordings of $[Ca^{2+}]_i$ of cells expressing Or22a and Orco stimulated with 100 µM ethyl hexanoate. Cells where the intensity of the first response was lower than 200 nM responded either to the first (A, n = 12) or to the second (B, n = 11) stimulation. Data represent mean ± SEM.

Supplementary Video. Example of Ca^{2+} imaging of an Or22a neuron expressing GCamP3.0. Left panel: raw fluorescence intensity data, the shown area is a magnified portion of the original dataset; right panel: ΔF/F (percent) of the left panel. The length of the calibration bar is 10 µm, the time interval between two frames is 5 seconds. Data analysis was performed in Fiji ImageJ version 2.0.0-rc-34/1.50a software (see Materials and Methods in the main text), video assembly was performed in Blender (Version 2.75a).
